# Medical News and Miscellany

**Published:** 1893-01

**Authors:** 


### Medical News and Miscellany.

A rare opportunity; see Dr. W. B. Anderson's card.

Dr. A. J. Weathered has removed from Osceola to Waco.

Dr. E. C. Dallas has removed from Alto to Ella, Brazos county, Texas.

Dr. Z. F. Lillard, of Tyler is stationed in St. Louis, at the city hospital, to which address he has ordered his JOURNAL sent.

In our February number will appear an excellent paper by Dr. A. E. Spohn, of Corpus Christi, illustrated by cuts; subject "Cœliotomy."

Dr. C. M. Ramsdell.—The JOURNAL is gratified to learn that since his removal to Eddy, New Mexico, Dr. Ramsdell has regained his health, which was impaired in consequence of La Grippe.

The Texas State Medical Association meets in Galveston, first Tuesday in May. Papers for section in surgery should be sent to Dr. A. B. Gardner, Chairman, Bellville. Dr. M. Smith, Black Jack Grove, is Secretary of the section.

Belford's Magazine.—The Christmas edition of this popular magazine was indeed a most creditable production. The reading matter is of a high order of excellence; and we dare say it is the ablest politico-literary journal published in America.

---

For Sale.—A four-room residence and two lots and practice worth $3000 a year for $1000, cash, in a flourishing railway division in the Panhandle country. Will sell the above property to any good physician, and start him in the practice. Address, Box 54, Clarendon, Texas.

---

Dr. B. M. Worsham, Assistant Superintendent Texas State Lunatic Asylum, at Austin, is visiting Northern and Western cities, and will examine the several systems of management of the leading lunatic asylums. Texas wants to have the best features of the best plans of doing exerything, and *will* have them.

---

State Health Officer Swearingen's biennial report is out. It shows the quarantine effectively and thoroughly administered, all diseases kept out or suppressed, and a balance on hand out of the none too liberal appropriation of $40,000. This is unprecedented, we believe. Of small-pox there were over 2000 cases in the State, with a mortality of 24.6 per cent. There is none now; not a case.

---

Mr. Jas. H. Bates, the popular, long-established and well-known journal advertising agent, announces that he has taken into his business his faithful manager and assistant, Mr. Lyman D. Morse, who has for the past three years practically had charge of the extensive business,—and the firm is now Bates & Morse, Advertising Agency, 38 Park Row, New York. The JOURNAL takes pleasure in recommending this agency, as prompt and reliable.

---

Its Fiftieth Year.—The *St. Louis Medical and Surgical Journal* announces the successful completion of its fiftieth year, and says, "To celebrate the event in a befitting manner, the issue of 1893 will be especially prepared." Its January number will contain portrait and biography of Dr. H. L. Linton, the founder of the enterprise, and during 1893 many excellent papers will be published and illustrated. Our subscribers can get the *St. Louis Medical and Surgical Journal* for $1, by adding that amount to their remittance for DANIEL'S TEXAS MEDICAL JOURNAL for 1893.

**Dr. W. H. Walker**, late of Ledbetter, has bought the property and practice of Dr. Harry C. Grace, at Oakland, Texas, and removed to that point. The JOURNAL regrets to learn that Dr. Grace is in bad health, which compels him to retire from practice. Both of these gentlemen are old subscribers and staunch friends of the JOURNAL.

---

**The Old Firm** of Burts, Field & Duringer, one of the oldest in Texas and a land-mark in Fort Worth, is dissolved by mutual consent. Dr. W. B. Burts, the senior partner, has associated his son with him, Dr. H. F. Burts, and from 1st January inst., this firm will be Drs. Burts & Burts. The JOURNAL has not learned further particulars, but assumes that Drs. Field & Duringer will continue to "do business at the old stand."

---

**Drs. Adams & Thompson**, of Fort Worth, a leading firm of general practitioners, and Medical Directors for Texas of the Equitable Life Assurance Co. have associated Dr. Bacon Saunders with them ; and the firm will be Adams, Thompson & Saunders. This is a strong combination,—all three being eminent practitioners. Dr. Saunders has long resided in Bonham and done a heavy practice, especially in surgery, in which branch he has made a brilliant reputation.

---

**Dr. T. J. McFarland**, a distinguished practitioner of Port Lavaca, Texas, formerly State Quarantine Officer under Gov. Ross' administration, spent several weeks in Austin in December and January. His son-in-law, Mr. J. R. Sheldon, of Austin, died early in December, and Dr. McFarland having been called to his bedside during his illness, remained to close up his daughter's business, and take her home with him. The doctor is an old college chum of the editor of the JOURNAL, and was a distinguished Confederate surgeon.

---

**Richard Fisher Bibb**, son of Dr. and Mrs. R. H. L. Bibb, of Saltillo, Mexico, made his initial bow on the stage of life at San Antonio, Texas, December 6, at 2:30 p. m., in the role of champion boy—fighting weight 10¾ pounds. The JOURNAL extends its most cordial congratulations to the happy parents, and hopes (and predicts) that in the fullness of time the President of these United States will bear the name of Richard F. Bibb. "Behind the cloud is the sun still shining," my dear doctor; may its rays ever warm and illuminate the pathway through life, of father, mother and son, is the wish of a host of attached friends. The storm-tossed vessel reaches haven at last, if manned by Captain Stout-heart and steered by Faith.

---

A valued correspondent in Chicago clips from our November number the remark that "Gov. Fifer, of Illinois, has appointed a homeopathic fledgling to be Surgeon General,—one on whose diploma the ink is not dry," and writes us that the profession of Illinois made an organized fight against Fifer, which resulted, last November, in the overthrow of his excellency, and the consequent downfall of his fledgeling Surgeon General. The latter will make his exit, along with "Private" Fifer, this month.

Our correspondent, further commenting on our remarks in same connection, says: "The disgraceful scenes at the White House [to which we referred] will also cease in a few weeks, and we will be honored by having a President who honors and respects scientific medicine, and has no use for quacks.

---

For Sale.—An opportunity to have a healthy, quiet and comfortable home, with a regular practice from the start of $2000 a year in a section of Texas that has the combined resources of the grain of the North, cotton of the South, and cattle of the West. Churches, school, Masonic lodge, cheap lands, a good opportunity to embark in the stock business. A bonanza for the worn-out practitioner of the malarial districts who is seeking health, and wishes to continue in practice. Improved acre lot, dwelling four rooms, two porches, chimney, cistern, garden, orchard, out-houses, and well, desirably located. Opposition weak. Terms, *cost*. Will remain till purchaser is thoroughly satisfied he can hold the field. Object, post graduate course. Address, W. B. Anderson, M. D.,
Content, Runnels county, Texas.

---

Death of Dr. Day.—Dr. Richard H. Day, of Baton Rouge, La., died on Sunday, December 4th (ult.) Dr. Day was born at Bladenburg, Md., June 9, 1813, and had he lived till June, would have been 80 years old. For a man of his age he was very active, and a zealous practitioner of medicine. He was devoted to his profession, and has left his mark on its literature, having contributed to the pages of several of the medical periodicals for many years. He had been president of his State Medical Society, and was at one time Professor of Diseases of women and children in N. O. Polyclinic. He was also one of the vice-presidents of the 9th International Medical Congress. As a man and a physician he ranked with the best, and was held in universal esteem wherever known. His was a busy and useful life, and the Journal hopes he has gone to the reward that awaits those who do their duty and improve the talent which God gave them.

**Confound the Printers!**—The JOURNAL received a *be-a-uti-ful* note from its esteemed friend and colleague, Wile, of the *N. E. Med. Monthly*, written in his well known clear, round chirography, and it being very important to the profession, gave it as "copy" to the printers; and just look what they made of it! It is too provoking! Anybody who has ever seen the doctor's handwriting can read it at a glance (but they don't know what he means):

DANBURY, Conn., De. 24, 1892.

*Lim Saul:*

Ipe eel exubub nite sissi ble ⌒‿ T z G. Monelz—Cohm *lu lu* che tree ale etc cobih 2. Of eye re—ie lb nomll os enfils *oo* I clo gr. duel! Cfu *nuece* c bun lun sleet buncombe z alce I t & m vin figllly ohu iley force grs. Io Tx— tonio r Ni 8 st coe PraeH. rocle.

W‿‿

---

The firm of Renz & Henry, Manufacturing Chemists, of Louisville, Kentucky, are justly held in high esteem by the Medical profession, and particularly by those in the South. By scrupulous attention to every detail of their business, and by a uniform courtesy in their intercourse with physicians, they have endeared themselves to the profession; and to-day there is no establishment, North or South, who has a better reputation for integrity, honesty and fair dealing than they. They have fairly earned the liberal share of Southern patronage bestowed upon them. They are indeed benefactors in the full sense of the word; and had nothing else been done to earn the title, the addition of their Elixir of Three Chlorides to the physician's resources would have won for them the gratitude of the profession. We take pleasure in directing the attention of our readers to their beautiful embossed advertisement, which ornaments this, the *Christmas Edition* of the JOURNAL, and in commending the house to all our readers as eminently reliable, honorable and worthy of the fullest confidence and respect. [Accidentally omitted last month.—ED.]

